# "I found that I was well and strong": Women’s motivations for remaining on ART under Option B+ in Malawi

**DOI:** 10.1371/journal.pone.0197854

**Published:** 2018-06-06

**Authors:** Nozgechi Phiri, Andreas D. Haas, Malango T. Msukwa, Lyson Tenthani, Olivia Keiser, Kali Tal

**Affiliations:** 1 Institute of Global Health, University of Geneva, Geneva, Switzerland; 2 Institute for Social and Preventive Medicine, University of Bern, Bern, Switzerland; 3 Baobab Health Trust, Lilongwe, Malawi; 4 International Training & Education Center for Health, Lilongwe, Malawi; 5 Institute for Primary Health Care (BIHAM), University of Bern, Bern, Switzerland; International AIDS Vaccine Initiative, UNITED STATES

## Abstract

Most Malawian women who start ART under Option B+ are still in care three years later, a higher than average adherence rate for life-threatening chronic disease treatments, worldwide (50%). We asked 75 Malawian on ART their motivations for remaining in treatment, and what barriers they overcame. Focus groups and interviews included 75 women on ART for 6+ months, at 12 health facilities. Four main motivations for continuing ART emerged: 1) evidence that ART improved their own and their children’s health; 2) strong desire to be healthy and keep their children healthy; 3) treatment was socially supported; 4) HIV/ART counselling effectively showed benefits of ART and told women what to expect. Women surmounted the following barriers: 1) stigma; 2) health care system; 3) economic; 4) side effects. Women stayed on ART because they believed it works. Future interventions should focus on emphasizing ART’s effectiveness, along with other services they provide.

## Introduction

In 2011, Malawi implemented an innovative policy (“Option B+”) to prevent mother-to-child transmission (PMTCT) by providing lifelong ART to all HIV-infected pregnant and breastfeeding women [1, 2]. Option B+ was subsequently adopted by other countries and is now recommended by WHO [3]. In Option B+ treatment programs, poor adherence, retention and loss-to-follow-up (LTFU) are still serious concerns, and continuation on long-term therapy is a critical element of HIV care [4]. We recently reported that 70% of the 29,313 women who started ART under Option B+ in Malawi by June 2012 were still in care three years later [5] and about 70% of the retained women adequately adhered during the first 2 years of ART [6].

If we take a global perspective on long-term therapy for chronic life-threatening diseases, Malawi’s Option B+ adherence and retention rates are impressively high, and much higher than in some other countries that have adopted the program. [7] Most papers on the Malawi Option B+ program examine retention and adherence, but do not put their findings into global context or compare them to retention in treatment programs for other chronic diseases. Poor adherence to long-term therapies and poor retention in programs that treat chronic diseases are common, and sometimes overlap [8,9]. The WHO defined adherence broadly in 2003 and found adherence rates to necessary medication are usually around 50% [10]. Even in clinical trials, the rate of patients who take prescribed doses of medication ranges from 43 to 78 percent [11]. Patients treated for chronic diseases may take inadequate or irregular doses of medicine, or they may stop attending treatment altogether (loss to follow-up or LTFU). Rates of LTFU are high for many treatment programs, including chronic myeloid leukemia (30–47%) [12], cardiovascular conditions (43%) [13], kidney transplant (28%), etc. Beginning with this premise, we believe the next logical step is to ask how Malawi can build on its success and successfully raise retention to an even higher level, and achieve the 2014 UNAIDS goals of ensuring that 90% of those diagnosed with HIV will remain on ART. [14]

Epidemiologists and public health experts who design and evaluate HIV treatment programs have studied the reasons for poor adherence to ART. Several systematic reviews highlight important individual-, interpersonal- and community-level, and health-system-level barriers to treatment uptake, retention in care, and adherence. On the individual-level, poor knowledge of HIV/ART [15,16], psychological issues following HIV diagnosis [15,17] forgetfulness [18], substance abuse [18,19], problems managing practical demands of ART [16,20], work- or family-related travel [18], and transport costs [17,20], are frequently cited barriers to adherence. HIV stigma [15,17,20,21], and fear of HIV status disclosure [15–18,20,21] are the most important interpersonal and community-level barriers. Limited accessibility to services and medication [15,16,18,19], poor staff-client interactions and staff attitudes [15,16,21], staff shortages [15,20,21], long waiting times [20], poor clinic practices and insufficient provider training [21,22], are frequently cited health-system level barriers to ART. Though motivation is a significant factor in adherence [11], we have little data on the range and intensity of motivations that drive Malawian women to adhere to ART in the medium- and long-term, and we know little about the interaction between women’s motivations for remaining on ART and their ability to navigate the complex network of barriers to treatment.

We visited 12 health care facilities across Malawi and conducted observations, semi-structured interviews (SSI), and focus group discussions (FGDs) with women retained on ART under Option B+ for a minimum of six months, to determine why they stayed on ART, and what barriers they had overcome to remain in treatment.

## Methods

### Study setting and population

This qualitative study was designed to complement our earlier quantitative studies on adherence and retention in Malawi’s Option B+ program [23,5,6,24], so we selected our study sites from the same list of facilities. We collected data from 12 facilities in the central and southern regions of Malawi (Table 1). At all 12 study facilities, women were initiated on ART in ANC clinics, but timing of referral of women from ANC to ART clinics varied depending on the model of care: [25] two facilities transferred women on the day of ART initiation; ten facilities transferred women at six weeks postpartum; and one facility transferred women two years after delivery (Table 1). We included a wide range of facilities because we wanted to identify patterns in retention common to women across different local cultures and regions, under different models of care.

**Table 1:** Characteristics of study facilities; number of semi-structured interviews (SSIs) and focus group discussions (FGDs) conducted at each facility.

Study facility	District	Location	Model of care	SSI (N)	FGD (N)s	Women in FGD (N)
Nsanje District Hospital	Nsanje	Rural	B	0	1	8
Bwaila District Hospital	Lilongwe	Urban	C	3	0	NA
Zomba Central Hospital	Zomba	Urban	A	2	0	NA
Limbe Health Centre	Blantyre	Urban	B	2	1	12
Machinga District Hospital	Machinga	Rural	A	2	1	12
Mchinji District Hospital	Mchinji	Rural	A	2	1	3
Salima District Hospital	Salima	Rural	A	1	1	7
Ntcheu District Hospital	Ntcheu	Rural	A	1	2	4+3
Phalombe Health Centre	Phalombe	Rural	A	0	1	7
Chikwawa District Hospital	Chikwawa	Rural	A	2	0	NA
Mulanje District Hospital	Mulanje	Rural	A	3	0	NA
Ntchisi District Hospital	Ntchisi	Rural	A	1	0	NA
**Total**				**19**	**8**	**56**

Model of care: HIV testing and counselling, initiation to antiretroviral therapy (ART), and adherence counselling is provided at the antenatal care clinic. Women transfer to the ART clinic 6 weeks postpartum (model A). HIV testing and counselling, initiation of ART, adherence counselling, and initial follow-up of ART is provided at the antenatal care clinic. Women transfer to the ART clinic on the day of ART initiation (B), or at 24 months postpartum (C).

We usually recruited participants at ART clinics, but some women were recruited from the ANC during routine antenatal visits. We included women aged 15 years or older who visited the facility and had remained on ART under Option B+ for at least 6 months. Health care workers (HCWs) helped us recruit our subjects by flagging patients who met our inclusion criteria and introduced us to them. We interviewed women and conducted FGDs until we reached data saturation (no new categories emerged in our data analysis, and no statement made by our participants could not be categorized [26]). We conducted both SSIs and FGDs because we wanted to hear from women under different social conditions. The SSIs provided privacy, so women could talk about subjects they might not want to mention in a group, and the FGDs promoted a relaxed, social, and open environment for women to share their experiences.

### Data collection

We triangulated our data, supplementing SSIs and FGDs with field notes from ethnographic observations. Both SSIs and FGDs used open-ended questions designed to elicit stories about motivations and barriers from participants. The subject’s narrative progress was guided by the interviewer, who then encouraged participants to expand on specific topics raised in their stories. The central question, to which the interviewer was instructed to return throughout the interview, was why participants stayed on ART long-term, and what challenges they faced in so doing. In the second part of the interview, the interviewer kept participants on track by asking them more probing questions about factors they and other participants said helped motivate them and allowed them to stay on ART. NP conducted all individual interviews and led all focus groups. We could not hold FGDs at every site because sometimes not enough women attended the facility on the day we visited. Sometimes enough women attended, but since we could only hold the FGD after all women were finished with their appointments with the nurse, some of these women would not wait for others to finish. Our observations focused on infrastructure (the layout and size of rooms), number of clients and health care workers, waiting times, the privacy and confidentiality afforded clients, and client-healthcare worker interactions.

We were aware that the level of privacy would change from facility to facility, and that we would need to be flexible and creative in our efforts to find or make comfortable spaces for participants to answer sensitive questions. Depending upon available facilities or weather, we held SSIs and FGDs in built environments, under a tree, or even in our vehicle if no more suitable place was available. Interviews were conducted in Chichewa, the local language, recorded on a Sony IC recorder, transcribed in Chichewa, and then translated into English by research assistants. NP, who is bilingual in Chichewa and English, vetted all translations for accuracy. Our researcher in the field (NP), accompanied our quantitative research team to health facilities, where she observed interactions and events in public areas of the hospital or clinic, and sometimes spoke casually to patients or HCWs in and around those areas. She took light notes while observing and followed those up with detailed field notes written in private. No confidential information was collected during the observations, and our field researcher kept no identifying records. Interviews were conducted between November 2014 and December 2015. We reviewed the literature, iteratively analyzed data, and reviewed ethnographic notes until late 2016.

### Data analysis

Our data analysis team consisted of a Malawian social scientist (NP; medical anthropology), and a Swiss-resident cultural studies scholar (KT; sociology, oral history, narrative theory). NP had the responsibility of coding transcripts independently in Atlas.ti. KT coded some transcripts in Atlas.ti and was responsible for closely reading all transcripts to identify narrative patterns. We took an inductive approach, reading and rereading the transcripts to identify key concepts in both SSIs and FGDs. We searched for patterns across the transcripts and grouped them into themes. NP and KT met several times to discuss emerging categories and themes, and then to finalize categories after saturation was reached. Disagreements were resolved by consensus.

We extracted data on characteristics of informants and mentioned barriers and facilitators from coded transcripts and generated quantitative datasets. We then used descriptive statistics to show characteristics of the informants who participated in FGDs and SSIs. We calculated proportions of informants who mentioned barriers and facilitators in FGDs and SSIs. We visualized patterns of barriers and facilitators that were mentioned in SSDs and FGDs in a matrix. The rows of the matrix represent barriers and facilitators, and the colons represent informants. Greyed boxes indicate that the informant mentioned a facilitator or barrier. Statistical analysis was done in Stata (Version 15. College Station, TX: StataCorp LLC).

We counted the barriers and facilitators mentioned in FGDs on the individual-level and not on the group-level. Participants often confirmed barriers and facilitators that were mentioned by someone else and we felt that it was not appropriate to count on group-level because views of individuals do not necessarily reflect the view of the whole group. We acknowledge that both approaches (i.e. counting on individual- and group-level) have advantages and disadvantaged, but we felt that the former approach was more appropriate.

### Ethical considerations

Participation was voluntary; patients were free to withdraw at any time without penalty. At the end of the interview or discussion, participants were given money to cover their transport to and from the clinic. We told potential interviewees about the goals of our study, after informing them about confidentiality, risks, and advantages. We explained we would use the information they gave us to publish academic papers, and we would share the results with the Ministry of Health. We included only subjects who gave us informed written or verbal consent, which we recorded at the beginning of each interview. In the FGDs, we assigned numbers to participants, and asked that they use the numbers instead of their names to protect their anonymity. These recordings were de-identified. After our research assistants transcribed the sessions, the interviewer (NP) carefully went over the transcripts again to ensure no personally identifying information was included. No other researchers handled transcripts before they were de-identified.

The study was approved in Malawi by the National Health Sciences Research Committee. In Switzerland, the Cantonal Ethics Committee of Bern waived the requirement for ethical approval for our study.

## Results

### Observations

NP observed at all facilities, for a minimum of 5 hours and wrote over 43,000 words of notes. Clinics always started with a health talk given by either expert client, a Health Surveillance Assistant (HSA) or nurse (student or otherwise), followed by HIV test and counseling, then antenatal care and ART. In addition to providing ANC, clinics offered services including family planning and cancer screening. Infrastructure was inadequate. In most clinics, there were not enough benches to seat all the clients. Some clinics did not have enough rooms to service their clients, and the rooms they had were often overcrowded and provided no privacy. NP noted that on most days the clinics had a large crowd of women and sometimes a few men who accompanied their wives. NP saw as many as 72 women attending ANC, and up to eight couples. Most of the clinics had a shortage of nurses. Sometimes one nurse handled the whole ANC clinic with the assistance of student nurses, expert clients and sometimes ward attendants (cleaners). Overcrowding and staff shortages meant women sometimes spent up to eight 8 hours at the facility.

### Patient interviews and FGD

We invited 80 pregnant or breastfeeding patients on ART to participate in our study. Of these, we ultimately included 75: 1 woman refused to participate; 3 waited for a while and left before the FGD started. We excluded one woman who was ineligible for the study from all analyses because she started ART long before Option B+ was introduced. Of the 75 women, 19 participated in the SSIs, and 56 participated in the FGDs. The median number of women per FGD was 7 (IQR 3.5–10). We conducted SSIs with patients at 11 out of 12 sites, and FGDs at 7 out of 12 sites (Table 1). Most interviews and FGDs were conducted at rural district hospitals, but we also collected data at an urban central hospital, district hospital and large health center. Interviews averaged about 30 minutes; FGD lasted at least an hour.

### Characteristics of study participants

Of the women who participated in FGDs and SSIs, two-thirds came from rural areas and the rest from urban areas. Most of the women were Christian, and most were Ngoni, Lomwe, Chewa or Yao. More than 80% of the women were married; more than half had finished upper primary or secondary school. Their median age was 30 years (IQR 24–33). Most were either housewives or small business owners and had 3 children. Participants in our SSI were on ART for a median length of 12 months at the time of the interview (IQR: 6–30). For more details on the characteristics of women who participated in SSIs or FGDs, see Table 2.

**Table 2:** Characteristics of women on Option B+ who participated in focus group discussions and semi-structure interviews.

	Focus group discussions N = 56 (74.7%)		Semi-structure interviews N = 19 (25.3%)		Total N = 75 (100.0%)	
**Residence**						
Rural	44	(78.6%)	12	(63.2%)	56	(74.7%)
Urban	12	(21.4%)	7	(36.8%)	19	(25.3%)
**Religion**						
Christian	49	(87.5%)	15	(78.9%)	64	(85.3%)
Moslem	6	(10.7%)	2	(10.5%)	8	(10.7%)
Unknown	1	(1.8%)	2	(10.5%)	3	(4.0%)
**Tribe**						
Chewa	8	(14.3%)	5	(26.3%)	13	(17.3%)
Lomwe	13	(23.2%)	3	(15.8%)	16	(21.3%)
Mang’anja	0	(0.0%)	2	(10.5%)	2	(2.7%)
Ngoni	15	(26.8%)	3	(15.8%)	18	(24.0%)
Nyanja	1	(1.8%)	0	(0.0%)	1	(1.3%)
Sena	7	(12.5%)	1	(5.3%)	8	(10.7%)
Tumbuka	3	(5.4%)	0	(0.0%)	3	(4.0%)
Yao	8	(14.3%)	5	(26.3%)	13	(17.3%)
Unknown	1	(1.8%)	0	(0.0%)	1	(1.3%)
**Time on ART (months)**						
6–12	0	(0.0%)	11	(57.9%)	11	(14.7%)
13–24	0	(0.0%)	3	(15.8%)	3	(4.0%)
25+	0	(0.0%)	5	(26.3%)	5	(6.7%)
Unknown	56	(100.0%)	0	(0.0%)	56	(74.7%)
Median (IQR)			12	(6–30)	12	(6–30)
**Age (years)**						
18–24	13	(23.6%)	6	(31.6%)	19	(25.7%)
25–29	14	(25.5%)	3	(15.8%)	17	(23.0%)
30–34	17	(30.9%)	7	(36.8%)	24	(32.4%)
35+	11	(20.0%)	3	(15.8%)	14	(18.9%)
Median (IQR)	30	(25–34)	30	(23–33)	30	(24–33)
**Number of children**						
0	0	(0.0%)	1	(5.3%)	1	(1.4%)
1–2	17	(30.9%)	9	(47.4%)	26	(35.1%)
3–4	26	(47.3%)	7	(36.8%)	33	(44.6%)
5+	12	(21.8%)	2	(10.5%)	14	(18.9%)
Median (IQR)	3	(2–4)	2	(2–4)	3	(2–4)
**Marital status**						
Married	49	(87.5%)	13	(68.4%)	62	(82.7%)
Divorced	3	(5.4%)	4	(21.1%)	7	(9.3%)
Separated	0	(0.0%)	2	(10.5%)	2	(2.7%)
Single	1	(1.8%)	0	(0.0%)	1	(1.3%)
Widow	3	(5.4%)	0	(0.0%)	3	(4.0%)
**Education**						
None	6	(10.7%)	0	(0.0%)	6	(8.0%)
Lower primary school	9	(16.1%)	5	(26.3%)	14	(18.7%)
Upper primary school	26	(46.4%)	6	(31.6%)	32	(42.7%)
Secondary school	14	(25.0%)	7	(36.8%)	21	(28.0%)
Unknown	1	(1.8%)	1	(5.3%)	2	(2.7%)
**Occupation**						
Small business owner	15	(26.8%)	5	(26.3%)	20	(26.7%)
Farming	9	(16.1%)	0	(0.0%)	9	(12.0%)
Housewife	18	(32.1%)	10	(52.6%)	28	(37.3%)
Salaried worker	6	(10.7%)	1	(5.3%)	7	(9.3%)
Piece worker*	7	(12.5%)	2	(10.5%)	9	(12.0%)
Unknown	1	(1.8%)	1	(5.3%)	2	(2.7%)

*Women doing short-term jobs

### Reasons women continued ART

Almost all women were aware that ART improved quality of life and likelihood of survival, for themselves and for their children. They shared their own experiences on ART, their observations of others, and the advice given to them by health providers or family members. As we coded the data, four major motivations for continuing ART emerged: 1) evidence they had collected from their own experience with ART or from observing others on ART, which convinced them that ART improved their own and their children’s health (35/75); 2) they had a strong desire to be healthy 40/75 and to keep their children healthy (34/75); 3) their treatment was socially supported (31/75); and, 4) HIV/ART counselling helped them understand the benefits of ART and what they could expect (36/75) (Tables 3 and 4).

**Table 3:** Facilitators and barriers to ART adherence and retention mentioned in semi-structured interviews (SSIs) and focus group discussions.

	SSIs																						SSIs & FGDs
	Rural													Urban								Total	
	1	2	3	4	5	6	7	13	14	15	19	20	n = 12	8	9	10	11	12	16	17	n-7	n = 19	n = 75
**MOTIVATIONS**																							
1. Empirical evidence in effectiveness of ART*																							
ART improves perceived health/physical function													9								4	13 (68%)	35 (47%)
Evidence ART prevents MTCT**													5								3	8 (42%)	27 (36%)
Evidence that ART works in friends or family													2								3	5 (26%)	13 (17%)
2. Protecting self and others from HIV***																							
Protect self													10								7	17 (89%)	40 (53%)
Protect baby/children													9								4	13 (68%)	34 (45%)
**3. Encouragement from others**																							
Family													9								4	13 (68%)	31 (41%)
Husband/partner													7								5	12 (63%)	42 (56%)
Health care workers’ counselling													7								6	13 (68%)	36 (48%)
Friends													2								1	3 (16%)	12 (16%)
**BARRIERS**																							
**4. Side effects**																							
Other drug side effects													9								4	13 (68%)	43 (57%)
Hunger													4								1	5 (26%)	19 (25%)
**3. Economic barriers**																							
Transport costs													6								3	9 (47%)	19 (25%)
Food insecurity													2								1	3 (16%)	6 (8%)
**1. Social barriers**																							
Fear of disclosure to partner/relative													6								3	9 (47%)	16 (21%)
Ongoing difficulties with partners													3								2	5 (26%)	12 (16%)
Stigmatization (including verbal abuse, exclusion)													1								3	4 (21%)	10 (13%)
Difficulties of chronic medication in everyday life													3								2	5 (26%)	7 (9%)
**2. Health care barriers**																							
Long waiting times													8								2	10 (53%)	32 (43%)
Abusive health care workers													3								1	4 (21%)	19 (25%)
Difficulties collecting sufficient drug supply													1								2	3 (16%)	5 (7%)

Data are numbers (percentages) of informants who mentioned a facilitator or barrier in semi-structured interviews or focus group discussions.

Greyed boxes indicate the informant mentioned a facilitator or barrier in semi-structured interviews

SSIs: semi-structured interviews; FGDs: focus group discussions

*Antiretroviral therapy (ART)

**Mother to child transmission (MTCT)

***Human Immunodeficiency virus (HIV)

**Table 4:** Facilitators and barriers to ART adherence and retention mentioned in focus group discussions (FGDs).

MOTIVATIONS	Rural																																													Urban													N = 56
1. Empirical evidence in effectiveness of ART*																																																											
ART improves perceived health/ physical functioning																																													17													5	22 (39%)
Evidence that ART prevents MTCT **																																													15													4	19 (34%)
Evidence that ART works in friends/family																																													7													1	8 (14%)
**2. Protecting self /others from HIV** ***																																																											
Protect self																																													21													3	24 (43%)
Protect baby/children																																													16													5	21 (38%)
**3. Encouragement from others**																																																											
Family																																													16													2	18 (32%)
Husband/partner																																													23													7	30 (54%)
Health care workers’ counselling																																													18													5	23 (41%)
Friends																																													9													0	9 (16%)
**BARRIERS**																																																											
**4. Side effects**																																																											
Other drug side effects																																													25													5	30 (54%)
Hunger caused by ART																																													13													1	14 (25%)
**3. Economic barriers**																																																											
Transport costs																																													10													0	10 (18%)
Food insecurity																																													3													0	3 (5%)
**1. Social barriers**																																																											
Fear of disclosure to partner/ relative																																													7													0	7 (13%)
Ongoing difficulties with partners																																													7													0	7 (13%)
Stigmatization (including verbal abuse, exclusion)																																													5													1	6 (11%)
Difficulties of chronic medication in everyday life																																													2													0	2 (4%)
**2. Health care barriers**																																																											
Long waiting times																																													16													6	22 (39%
Abusive health care workers																																													12													3	15 (27)
Difficulties collecting sufficient drug supply																																													2													0	2 (4%)

Each color represents an FGD, listed in order of their identity numbers. Filled boxes indicate an informant mentioned a facilitator or barrier in the FGD.

*Antiretroviral therapy (ART)

**Mother to child transmission (MTCT)

***Human Immunodeficiency virus (HIV)

#### 1) Empirical evidence

Many women (35/75) explained that they stayed on ART because they noted that they had less or no illness since they started.

“… as for me I do not find any problems ever since I started… and I have never been sick up to coming to the hospital to be admitted.”

32-year-old, FGD003

After their children were born, most of these women were motivated to continue ART after their child’s HIV test was negative (27/75). They anxiously waited for all three post-partum HIV tests (6 weeks, 12 months, and 24 months) to come out negative. Every negative result was proof that the drugs were effective.

Because this child, her sibling taught me a lot till now. I got pregnant while still taking [ART]. Until her birth. After 6 months, I came again to get her tested for the first time. After one year and some months, I came again for her to be tested and the results were the same, she was HIV-negative. After 2 years and 2 months, I came for the last time, her blood was the same [HIV-negative]. For sure that is the goodness that I see.”

33-year-old, SS1004

Women also spoke of being inspired by evidence that ART had worked for some people they knew. They mentioned family members or friends who were on ART for some time and are healthy and doing well (13/75).

“I am motivated… I have my aunt who was found HIV-positive. She had twins, a girl and a boy. They got sick and they went to the hospital and they were tested…. They found that the boy was HIV positive and yet the girl was HIV negative. Now my child is 1 year 6 months and that child is 12 years and still taking the medication together with my aunt… That is what motivates me that ah how is she staying and this small child… he has stayed all these years….”

28 years old, SSI010

#### 2) Strong motivation to keep themselves and their children healthy

Almost all women said that they took ART to protect their own health, and to protect their children from contracting the virus.

“But also, I was sick and sometimes I would say ah! But now it’s not okay, but after I started receiving the drugs I found that I was well and strong, and I can walk with ease. So, since that time to now I am okay.”

24- year old, FGD005

“Yes, so that maybe the baby should be born well. HIV-negative right? If you stop, have you loved your child? …I continue, and we are continuing… really…”

30-year old, SSI008

Some women said they took ART to live long enough to see their children grow, because no one else could take care of their children if they died prematurely.

“… I see nobody who will take care of my children. So at least when I am dying, if I am to die… That is if the world does not end while I am alive, I should see how my children’s future will turn out. That’s why am still taking the medicine daily.”

30-year-old, SSI008

The realization that their child’s health was in their hands motivated them to stay on ART even when they experienced side-effects.

#### 3) Social support

Most of the women we interviewed (65/75) said they had gone to ANC and were tested on their own. Almost every one of them went home and told their partner of the test result, most of them the same day. One waited a few days, but eventually told her partner. Only two women said they had not disclosed to their partners. Most women said their partners accepted the result and agreed to be tested.

“When I came to start antenatal, they said I should come for testing, so after being tested I was found HIV positive. Then I went home and explained to my husband. I told him that we should go to hospital together for testing. I have already found out my status, am HIV positive. You should also go for testing and we went. He is also HIV positive”

26-year-old, SSI003

Some of the women said they received support from their partners even though their partners did not get their own HIV test.

“He never came for the test. I would lie if I say he came. But he never went…. He just encourages me to be taking [ART] so that my child will be born without the virus.”

33-year-old, SSI002

Some women (10/75) went to ANC with their partner and were tested together. Those with discordant results (8/75) rarely had arguments with their partners or were insulted by them. Instead, their partners encouraged them to continue treatment.

“Before they told us the results with my husband, they asked us a few questions. They asked him if I tested positive could he accept… can he continue being with me or not? He accepted that he would stay with me. They asked me as well. I said I would be with him still, so he accepted, and we are still together”

29-year-old, FGD001

Only once during our observation did we hear of a marriage dissolving after a discordant result on previous day. Most women spoke of being encouraged by their partners. Others mentioned that their partner would sometimes collect their medicine for them or remind them of their appointment dates.

“Yes, my husband was receiving the medicines for me and bringing them to me.”

24-year-old, SSI0012

“No, he has never denied me and also that time he had the heart of reminding me about the ARVs.”

29-year-old, FGD002

Most women disclosed to and were encouraged by one or more family members (31/75) or partners (42/75). Usually, they chose to tell their mothers. Those who did not have a good enough relationship with their mothers disclosed to other family members, including sisters, uncles, and children. Very few women disclosed to their friends, but the few that did said these friends were also on ART. Those who disclosed to family, partner, or friends said they received encouragement to continue treatment, to take pills without skipping doses, and to keep their appointment dates.

“When I went home, I told all my relatives, we gathered and discussed. So, people came, ‘don’t worry you shouldn’t be anxious. It’s better you listen to the counselling you receive from hospital’.”

39 years old woman, SSI0019

“… there was my friend whom I chatted with. I didn’t know that she already started taking ARVs. I explained to her and she said, ‘there is no problem, I will be your witness [guardian] just like your relative’”.

36-year-old, FGD007

#### 4) HIV/ ART counselling

Almost all the women were worried after being diagnosed with HIV. Some women were anxious about taking the drugs every day for the rest of their life. Others worried about infecting their unborn child. Some women worried that their partner would not respond well to the news they were HIV-positive. Many of these women’s fears were allayed by counseling. Most (36/75) appreciated the counselling they received from the health providers, on the very first day or thereafter. They felt that counseling helped them overcome these worries and helped them stay on ART.

“I was stressed about ah! I will be taking ARVs every day. I was also worried about the unborn child, that the baby would be infected, and it would be difficult for me to take care of him. So, the nurse explained to me that ah! When you follow what we will tell you here, your child will what? Will be okay, mm hm!”.

29-year-old, SSI002

“… when I was diagnosed with HIV, when I went for counseling they said that, when you start taking the drugs it is not good to stop, the minute you stop taking the drugs your life is never okay …”

22-year-old, FGD005

Most women experienced drug side effects like dizziness, heart palpitations, numbness, nightmares, vomiting, a feeling of drunkenness, and extreme hunger. Women mentioned that HCWs helped them get through the side effects and observed that side effects diminished or disappeared, just as medical personnel had assured them.

“… the people that were teaching us said that when one has just started taking ARVs, they said she can experience some kind of signs. Maybe if you take the drugs, maybe you feel dizzy, maybe the heart beats fast, or maybe people feel numb… they said so. When the ARVs have adapted, well, it all stops. And when I started taking them, it happened to me for a week then it stopped. Now I don’t have any problem. I saw that the hospital staff were saying the truth.”

30 years old, FGD007

“I should say, when I was 5 months pregnant, that is when I decided to go to the hospital. They asked me why were you not coming to get more ARVs all these months? I told them that I was scared to take the ARVs. It’s not like they are scary but why were you not taking the drugs? I told them that they are making me feel drunk. They are making you drunk? Eh! You should go and drink them continuously because the child you are going to have can be infected with HIV, and he cannot live long, and you cannot live long too. That is what forced me to start taking the ARVs as advised.”

19-year-old, FGD002

In some areas, women said that community health workers (CHWs) followed them at home. These follow-up visits focused on their adherence to the ARVs, and side-effects of the drugs. Most appreciated the encouragement they received from these CHWs.

“Yes, I have accepted that they used to follow me… he used to give me very good counsel, and we used to chat very well, about how things are. He would even help me get the drugs, help me count them, and tell me how it is. When he came here, I would always find peace in my heart, because we were chatting very well.”

29-year-old, FGD002

### Barriers to retention

All the women we interviewed mentioned barriers to retention–obstacles that they overcame either singly or repeatedly, to continue treatment. We divided these barriers into four groups: 1) social barriers; 2) health care barriers; 3) economic barriers; 4) side effects of medication. These are described in detail below and in Table 3.

#### 1) Social barriers

Disclosure is an immediate and pressing issue for women diagnosed with HIV on Option B+, especially since HCWs ask women who test HIV positive to identify guardians who can support them in their treatment and bring those guardians to their next ART visit. All the women we interviewed had disclosed to a partner, family member or friend. Among the women we interviewed, disclosure to their partner often did not have the consequences they feared.

“I went to the village, so I left the drugs… I took some and left some at home. So, when I left them like that, he found them when he was left in the house so when I came back that is when he was asking ‘what are these drugs for?’ So, I then told him that those drugs I was given at the hospital. Why he said? Then I started explaining that I was tested, and I am HIV positive. So that’s when he said alright. Why didn’t you tell me all this time? I said I was afraid. He said do not be afraid….”

40-year-old, FGD005

Some (12/75) of the women we interviewed had ongoing difficulties with their partners. Sometimes partners (5/75) denied they could be HIV-positive, accused their wife of bringing the infection into the house, and provided no support.

“When I went home I explained it to my husband. My husband answered that ‘no, that means you are the one who went and contracted the infection,’ and so on….”

28-year-old, FGD004

Some women hid their clinic appointment from their spouse to avoid quarrelling about the infection. They mixed their clinic visit with some business they had in town, so they could give their spouse a different reason for being in the area.

“A while back like I said I would say I am going and when I come back we would quarrel about many issues. He would say so many things. Then I saw that I should not be troubled. I would make it so that when I take my business, he will be saying ‘my wife has gone to do business’. Like now, I have left it with someone. Maybe I can meet him he would say ‘what is it?’ ‘Oh, I am coming from the market. I went to feed the child cornmeal pulp.’ After that we go our separate ways”

36-year old, SSI009

A few women we interviewed were stigmatized by partners, families, and community members, and dealt with abuse on a regular basis, ranging from verbal attacks to exclusion.

“But when we are arguing at home, they say that ‘this one is already finished, she takes ARVs, and she is finished…. She wants to give us bad luck… she is running on foam.”

32-year-old, FGD004

“So, this other time we quarreled and my husband went outside, ‘oh, you dog, you are stupid, and you should live with your infection.’ Then I went into the house and started deliberating. I was alone and then I said ‘oh, my relatives here should know about this, I should not hide it from them.’ I explained it to my relatives. Then it happened that I have been taking the medication from that time up to now, but I dig and bury them, because when we quarrel he takes out the bottles and throws them outside. So, I felt that I was being embarrassed at the plot.”

36-year old, SSI009

#### 2) Health care barriers

Women talked about long waiting times at health facilities, which were usually very crowded. This forced them to leave home early, so they ensure they were near the front of the line for treatment. Some women said waiting times were longer because HCWs were overworked or distracted with activities unrelated to work, that clinics were short staffed, or that HCWs did not open on time. Some complained that certain HCWs practiced favoritism.

“I have concerns… the number of nurses during children’s clinic…. that is what concerns me because you find that you have left some chores undone at home that aah! Maybe when I come back from the hospital I should tend to them and you find that you take forever here at the hospital, that means you won’t do those chores because you get home late, yes.”

24-year-old, SSI0012

“You find that some of the staff here take health passports of people outside and receive the drugs for them while you are just standing in line, you wonder what is happening, you hear eh! He is helping those people first maybe 2 people. That is quite worrying because some of us come here early in the morning we stay here all morning …”

37-year-old, FGD005

We also documented understaffing in our field notes:

“…the nurse told me that she was the only one on duty doing the clinic. She was being helped by the student nurses. She explained that the department has four nurses. Two were out for workshops and one was doing cervical cancer screening in another room. She started complaining that the clinic has stalled because there is no one in the PMTCT room to do the testing.”

(Field notes, NP, 24-07-15)

“The nurse complained that the day has been hectic and long… She saw about 86 clients by the end of the clinic and the majority which she saw herself. She did the best she could to be fast, but it would have been better if there were at least 2 nurses in that room. She was also not able to spend so much time with each client that was initiated on ART.”

(Field notes, NP, 08-11-15)

There were women who reported abusive treatment from HCWs, including being yelled at. We also documented shouting and HCW frustration in our field notes, though it was infrequent in our presence.

“Most of the women didn’t know their ages. The counsellor was getting frustrated with every little thing, if the woman didn’t answer quickly or didn’t know how far along she was. I could hear her frustration in her voice. She was somehow scolding the women every now and again… she complained that she was already tired”

(Field notes, NP, 14-12-15)

Women who missed their appointment dates or whose pill counts did not match medical expectations were sometimes denied drug refills or sent to the back of line and usually treated as if they didn’t understand the importance of ART. They often had to attend group counselling again, which made their day at the clinic longer.

“I don’t know what kind of mistake they made in the bottle. The ARVs run out before my next appointment to receive ARVs. When I came here and explained they took my card [ART medical file] and threw it outside saying, ‘You won’t receive ARVs. It means you were sharing them with your friends’. So, I just sat there and didn’t say anything. [*I*: *Weren’t you disappointed that you thought of stopping right away*, *with the way you have explained*?] No, I wasn’t disappointed, I came because it’s my life not hers, I came.”

36-year-old, FGD007

“I have been encountering problems a few times, you come here they do a pill count and they tell me that I skipped one day, and then they put me at the back of the line, so that everyone else should be served and I should be served last because I skipped one day…. now you are saying all these people should go in and I should be waiting for them to go in even though I was the first person to show up here … So that is where maybe the doctors don’t do right by us.”

FGD002, 19-year-old

“So, if you missed [your appointment date] by a long time they tell you to go for group counselling, it means you did not learn well. So, they also take the guardian that accompany us… they call him/ her again, on Thursdays, on Fridays, I think. We go for counselling together, with the aim that she should encourage me to come every day of my appointments. …”

(FGD004, 32 years old)

#### 3) Economic barriers

Two economic barriers stood between some of these women and continuous ART treatment, and the effort to overcome them was continuous. The first was transportation, a barrier for a quarter of the women we interviewed (19/75). The second, mentioned much less frequently, was food insecurity (6/75). Food insecurity is common throughout Malawi; in April 2015, the World Food Program conservatively estimated that over 2.85 million people were food insecure (about 16.5% of the population) [27]. Food insecurity increases and decreases with drought conditions but is always higher among women [28]. We provide quotes to document food insecurity under “Side effects,” in the next section, since food and side effects are almost always mentioned in tandem.

Most of the women walked long distances to get to the hospital, if they did not have enough money to pay transport fees. Nineteen women mentioned walking for more than an hour to get to the facility. Those who did not have their own bicycles and were not dropped off by their partners did not regularly have money to pay for transport costs, though a minibus was available if they lived in urban areas, or a bicycle taxi in rural areas. Women went to health facilities far from their homes for different reasons. For most women, there was no closer health facility. One woman chose a far-away health facility because it was close to a market, and she combined the visit with a trip to the market for either business or shopping and used it as an excuse to keep her hospital visits a secret. Some women preferred attending the district hospitals (a “real hospital”) to attending local health centers.

“Yes, it takes us time like that because we are coming from far. Because we leave our homes maybe before four, because our homes are very far. So, when we have no money for bicycle taxi they charge 500. When we have no money for transport we just know that we will wake up before four and so when we get here maybe 5 o’clock. So, to be here till now is something that worries us a lot, because the way it is the time has gone,”

29-years-old woman, FGD002

“I have come with my husband he dropped me by bike. He is gone… He has left me 200 Kwacha for me to use when going back it will not be enough, the other side I will walk”

26-year-old woman, SSI003

Sometimes, because of the distance women missed appointments, but they still visited the health facility before their pills ran out.

“So, a person is coming from… maybe you are coming from all the way so for you to get here, on foot, you have no transport. Getting here becomes difficult. So, it can happen that maybe… you can miss the appointment day, thinking how am I going to travel? You become lazy, right?”

36-years-old, FGD004

The first 6 months after ART initiation and after giving birth, women are expected to come every month for their drug refills so that the HCWs can monitor their adherence and their baby’s health/growth. Thereafter, based on their adherence trend, women are given refills at 2- or 3-month intervals. Most women preferred the longer intervals over the monthly visits.

“But previously we were at pains that we were coming every month, when the month ends. It’s better now the government considered us by giving us medicine for 3 months, we do rest.”

30-years-old woman, SSI007

#### 4) Side effects

Most women (43/75) said they had had side effects from ARVs in the first few weeks after they started treatment. Side effects included dizziness, numbness in the feet, heart palpitation, nightmares, a feeling of lightheadedness, and excessive hunger. A few women admitted they had stopped taking ARVs for a short period because of the side effects. The side effects frequently disappeared or diminished if women took their ARVs at night before bed, ate sufficiently, and continued their ARVs without fail for several weeks. The one complaint that persisted was hunger. Women (19/75) emphasized that eating sufficiently was necessary to prevent them from feeling weak or sick in the morning.

“…the reason why I stopped taking medicine was one, food was a problem. In terms of food stuffs… It was difficult for me. When I eat, it was like I haven’t eaten any food, and my heart was like… Pounding…”

22-year-old, SSI020

### Differences between rural and urban sites

We did check for differences between rural and urban sites and found women’s responses were more similar than different. The women at urban sites mentioned transport costs and long waiting times in health facilities much less often than women at rural sites. Women at urban sites talked more about fear of disclosure than women at rural sites.

## Discussion

We found that Malawian women made the decision to remain on ART based on their own desires and priorities. They wanted to keep themselves and their children healthy, and they saw evidence that ART improved their health and the health of their children. Their treatment was socially supported by partners, family or friends. HIV counsellors helped them understand the importance of treatment and encouraged them to continue; they also explained the likelihood of side effects and told them how to cope with them. All the women we interviewed mentioned overcoming some barriers to treatment, most commonly side effects, social barriers (primarily related to disclosure and stigmatization), long wait times, and difficulty with transport. Most women had similar motivations, but there was wider variation in the barriers they described. Results were similar across SSIs and FGDs and matched our observations.

We discuss our limitations first, and then place them in context. We interviewed women retained in care, and not those lost to follow-up (LTFU). We could not compare the presence or absence of motivations and barriers between women who continued and discontinued treatment. For example, we found women were motivated to stay on ART when they saw evidence it was effective, but we could not determine if the perception that treatment was ineffective caused women to drop out. We did, however, have access to other studies that considered the motivations and barriers faced by HIV-positive women who spent less than 6 months on ART, and by women LTFU.

Zhou (2016) and Kim, et al. (2016), for example, interviewed a group of recently diagnosed women with HIV at health care facilities in Lilongwe [29,30]. Though their samples did not represent all women on ART in Option B+ in Malawi, these studies captured most of the same motivations and barriers earlier studies had identified. Some of their findings were conspicuous by their absence in our study, including skepticism about starting and continuing lifelong treatment when asymptomatic, the belief that counseling didn’t provide enough information, and discomfort with starting ART on the day of diagnosis. In Zhou’s study, women sometimes didn’t trust their diagnosis, but in our study, women did not express doubt. No woman we interviewed said that she had been retested or sought a second opinion. It is useful to think of these two studies as describing one end of a funnel, which includes, at its mouth, every woman identified as HIV-positive in the Option B+ program. Our study population represents the smaller group of women (67%) who made it through the relatively wide throat of the funnel.

The women we interviewed did not face insurmountable structural barriers to treatment, decided to stay on ART, and had the resources to carry through their decisions. Our study and Zhou’s also represent two different time points in women’s ART treatment. Unlike Flax et al., we did not include women lost to follow-up; otherwise, the pool we drew from was similar. Though findings about barriers to ART should not be generalized to women lost to follow up, Flax et al. did report that women lost to follow-up faced similar barriers, including side effects, costs, and poor treatment by health care workers [31].

Our study was strengthened by our use of multiple data collection techniques: we gave women opportunities to share their experiences in either SSI or FGD), and we collected observational data that augmented the data we collected in interviews and group discussions. For example, we observed the challenges health care workers faced in providing women with privacy and keeping their data confidential, though women did not discuss this much in the interviews. Our results were also consistent across the three methods of qualitative data collection we used: SSIs, FGDs, and observational notes. The cross-sectional nature of our study prevented us from examining how women’s barriers and motivations shift over the course of lifelong ART, though we suspect they do. Younger women were underrepresented in our study, which may be explained by our quantitative team’s finding that women in the first 6 months of ART treatment are most vulnerable to LTFU, while women retained for over a year default at a much lower rate [5]. Our study was strengthened by the number of women it included, and the fact that our informants were drawn from diverse regions and health facilities across Malawi, but this coverage was not complete. We included only a few women from tribes that mainly reside in the northern region of Malawi and our findings may not be transferrable beyond Malawi’s central and southern regions.

Most women we interviewed explained that they continued treatment because they saw evidence that ART improved their own and their children’s health. Women who stayed in care agreed that ART reduced morbidity and increased energy for chores like farming. Their beliefs in the effectiveness of medication were based on their own experience of taking medication that reduced symptoms or caused side effects, and on observing other patients who started, continued or stopped the medicine. Our findings are in line with those of earlier studies on medication adherence of patients on long-term therapies for the chronically ill. When patients do not feel better after treatment, they take it as evidence the treatment is ineffective; patients also see drug side effects as evidence a drug is harmful [9,32,33]. In general, patients value their own experiences above what doctors tell them and decide whether they will take a medication based on their personal assessment of the therapy [9,34]. Studies on ART patients from sub-Saharan Africa reported similar findings. Van Loggerenberg et al. found that ART patients were motivated by evidence of change in themselves, or in other people who were sick and grew healthy again. [35] Watt et al. found that HIV patients adhered well after their health improved considerably after ART start. [36] Katiri et al. found that women were encouraged by how well their friends on ART looked. [37] Elwell et al. also found that women were motivated by seeing other people on ART survive, and continued ART so they could see their children grow. [38]

Because most women in our study were in antenatal care, our finding that women were motivated to stay healthy to care for their children was not unexpected. Earlier studies found that women did not want to pass the virus to their child, either during or after pregnancy. [39,29] We found that women who were having, or had just had their first child on Option B+ were more concerned about passing on the virus than women who had already given birth on Option B+. Once women bore an HIV-negative child, they grew confident that ART had a protective effect. Women’s belief in the effectiveness of ART was reinforced each time the child’s HIV test was negative (i.e. at six weeks, one year, and two years after birth). In our interviews, women continued to be concerned and anxious about their child’s HIV test results and were relieved at a negative result. This supports Bezabhe et al’s finding that women were determined to follow instructions and adhere throughout their next pregnancy. [39] An increasing belief in the effectiveness of ART may help explain lower rates of loss to follow-up and good adherence among those retained in their second and third years on ART. [5,6]

Almost all the women in our study disclosed to their partners and asked them for advice and support for ART, but in cases where they did not receive support, or even received negative feedback, the women we interviewed were not discouraged. They began ART because they believed it was best for them and their children, this aligns with Kim et al.’s finding that women seek their partner’s advice about whether they should continue ART, but then make their own decision. [30] We found that some women made independent treatment decisions in the face of partner indifference or opposition. Women without partner support found support in family members, and sometimes in friends who were already on ART. There was no woman in our study who said she received no social support at all. Even women whose partners had sero-discordant results said their partners accepted and supported them. Our informants defined external support as acceptance of their serostatus, sometimes combined with treatment support (reminders to take medication, encouragement to adhere and keep appointment dates). None of them said they received monetary support, and several spoke of themselves as poor or penniless. We heard no reports of physical abuse and heard only one mention of a marriage dissolving (in our observations, but not in our interviews). These may be rare events among HIV-positive women retained on ART.

Studies and systematic reviews have already identified many potential individual-level, interpersonal-level, community-level, and health-system-level barriers to adherence to ART. Our data showed that frequently cited health-system barriers (except long waiting times and transportation problems) have generally been addressed by Option B+ policy. Our participants frequently mentioned drug side effects and food insecurity when we asked them what made it difficult to adhere to ART. Side effects may crucially shape beliefs about the effectiveness of a drug, especially among asymptomatic HIV patients who do not feel “better” when on ART. Side effects were very common among the women that we interviewed, a finding confirmed by other studies from Malawi, [30,40] Women enrolled in the Option B+ program in Malawi received a daily fixed-dose combination containing efavirenz, tenofovir, and lamivudine [1]: a regimen known to cause moderate central nervous system side effects such as dizziness, insomnia, impaired concentration, somnolence, and abnormal dreams in about 50% of patients. These side effects begin soon after start of therapy, but usually resolve after several weeks. [41] A recent study from South Africa confirmed the very high prevalence of side effects among Option B+ women who started ART on efavirenz, tenofovir, and lamivudine during pregnancy. Almost all women reported experiencing at least one side effect and almost half of the women had more than five side effects. [42] Side effects were strongly associated with self-reported non-adherence to ART. [42] Like Flax et al, we found hunger caused severe side effects, suggesting food insecurity might be an important barrier to adherence. [43] We also observed barriers posed by stigma and disclosure, which aligns with a 2013 global systematic review and meta-synthesis of the effect of stigma on adherence to ART: HIV-related stigma posed barriers to treatment by “undermining social support and adaptive coping.” [44] The Lancet Commission’s 2015 report on HIV confirmed this, emphasizing the importance of addressing social problems in HIV treatment, including “stigma, discrimination, gender inequality, punitive laws, and other drivers of HIV transmission”. [45]

Several targeted interventions designed to improve ART retention among pregnant or postpartum women have been or are currently being tested in sub-Saharan Africa. [46] These include mobile health applications [47–50] integrated care models [25,51–53] peer counseling [54–56] and involving male partners. [57,58] There is evidence that phone-based interventions can improve retention, but the success of integrated care models has been variable. [46] The effectiveness of peer-supporters and treatment models that involve male partners is still under evaluation. [46] But none of these interventions is intended to directly alter women’s perception of ART medication and treatment.

Since hunger exacerbates side effects, and food insecurity is common in Malawi, Option B+ might be able to retain a higher number of food insecure women if interventions provide food supplements to those women who need them. Prescribing alternative first-line regimens (e.g. integrase inhibitors) might reduce other side effects. Since most of the women in our study said they found it helpful when HIV counselors warned them about side effects in advance, and advised them on the proper intervals and conditions for taking their medications, providing more and better counseling on ART and its side effects could raise the number of women who start and continue ART.

## Conclusion

Our own and other data demonstrate that, in the absence of insurmountable barriers, women’s perceptions of ART determine their treatment decisions. Future interventions should, along with whatever other services are provided, be aimed at directly changing those perceptions in ways that support the choice to continue ART.

## Supporting information

S1 Text Codebook. (DOCX)

S2 Text Observation guide. (DOCX)

S3 Text Semi-structured interview and focus group discussion guide. (DOCX)
