# Supplementary material for: "I found that I was well and strong": Women’s motivations for remaining on ART under Option B+ in Malawi
Source: PLoS One. 2018 Jun 6;13(6):e0197854. doi: 10.1371/journal.pone.0197854 (PMC5991368; doi:10.1371/journal.pone.0197854)
Supplement: S1 Text — (DOCX) [file pone.0197854.s001.docx]

**“I found that I was well and strong”: Women’s motivations for remaining on ART under Option B+ in Malawi [PONE-D-17-27063] [EMID:379ee272e0420312]**

**Codebook**

| **Codes** |  |
| --- | --- |
|  | **MOTIVATORS** |
| **Empirical evidence in ART** | **Definition:** The stories women shared on how they know ARVs work. The stories were from seeing others survive on ARVs or seeing a negative or positive test result of their latest or previous child.    **Subcodes:**  **ART improves health**- Women’s belief that ARVs has improved their health and body stamina  **ART prevents MTCT**- Women talked of negative HIV status of their children after taking the ARVs during pregnancy and breastfeeding  **Evidence ART works in friends or family**- Success stories of relatives or friends on ARVs that have survived on ART for years |
| **Protecting self and others** | **Definition:** Taking ARVs to prolong life to be able to take care of their children but also to prevent infecting their unborn or breastfeeding child.    **Subcodes:**  **Protecting baby-** Women’s perception that taking ARVs continuously would prevent them infecting their child  **Protecting self**- Women’s belief that taking ARVs would improve their immunity, would prolong their life |
|  |  |

| **Encouragement from others** | **Definition:** Any mention of encouragement from husbands, relatives or friends towards drug adherence or their HIV positive status  **Subcodes:**  **Family support-** Any mention of encouragement from relatives on HIV status, hospital appointments and drug adherence  **Husband support-** Any kind of encouragement from husbands towards drug adherence or HIV positive status  **Health care workers support-** Counseling from the health care workers either at home or at the health facility that women felt improved their understanding or awareness of their HIV status, drug adherence  **Friend support-** Encouragement from friends to start or stay on ARVs or acting as treatment supporter |
| --- | --- |

**BARRIERS**

| **Side effects** | **Definition:** Side effects that women experienced at the start of their ARVs. A few continued to feel them  **Subcodes:**  **Hunger side effect-** Severe hunger after taking ARVs or vomiting if taken without food  **Other side effects-** All other side effects women experienced from taking ARVs that do not include hunger such as dizziness, vomiting, nightmares |
| --- | --- |
| **Social barrier** | **Definition:** Any challenges women faced at home or in the community related to their HIV status  **Subcodes:**  **Fear of disclosure-** Women shared stories of fearing their husbands and relatives reactions of knowing their HIV status.  **Husband challenges-** Problems women continuously faced after disclosing their HIV status to their husband e.g. blame or harassment  **Stigmatization-** Relatives or friends mocking or laughing at the women's HIV status.  **Chronic medication**- Concern women shared about taking ARVs all their lives  **Blame for infection**- Women whose husbands accused them of bring the HIV infection in their house |
| **Economic barrier** | **Definition:** Women’s challenges with money.  **Subcodes:**  **Transport costs-** Women’s experiences getting to the health facility amid insufficient funds  **Food insecurity-** Women’s challenges taking ARVs when they did not have enough food in their house |
| **Health care barrier** | **Definition:** Challenges women faced at the health facility interacting with health care workers and problems getting treatment.  **Subcodes:**  **Waiting times-** The length of time women spent at the clinic from the time they arrived to when they left for home and their perception of why it sometime took so long.  **Abusive health workers**- Observations of health care workers shouting or showing frustration as they provided the service. Also ill treatment from the health care workers experienced by women  **Difficulty collecting drugs-** Women who failed to collect the ARVs for different reasons |
| **Couple testing** | Women that went through HIV testing with their husband |
| **Discordant couple** | Women whose husbands tested HIV negative |
