# Supplementary material for: "I found that I was well and strong": Women’s motivations for remaining on ART under Option B+ in Malawi
Source: PLoS One. 2018 Jun 6;13(6):e0197854. doi: 10.1371/journal.pone.0197854 (PMC5991368; doi:10.1371/journal.pone.0197854)
Supplement: S2 Text — (DOCX) [file pone.0197854.s002.docx]

**“I found that I was well and strong”: Women’s motivations for remaining on ART under Option B+ in Malawi [PONE-D-17-27063] [EMID:379ee272e0420312]**

**Observation guide**

Our observations in facilities will be at three different levels even though the lines separating these levels are not clear: (1) facility; (2) individual; and (3) interactional levels. At facility level, focus will be on the general structure of the facility and specifically the set-up of all the sections that are part of the Option B+ services. Our interest is on things like waiting space, number of staff and availability of equipment and drugs among others. At individual level, we will follow some clients (one at a time) from the time they arrive at the facility up to the time they leave. The idea behind is to understand all the processes and or stages that clients who are under the option B+ go through at the facility. Emphasis will be given to the total time spend at the facility, time spend at each station, the type of care or treatment they receive and who exactly accompanies them. We will make an effort to ensure that we have followed both new entries and those coming for follow up visits. Since observational method can also involve asking questions where necessary, we will briefly ask some women informally about their visit. In addition, we are also interested in the client – clinician interaction at the facility. As such, we will observe as much interactions as possible between the two. We will focus on the mood, feedback, content and specific words used among others. This will help us to understand the relationship between the two and how that might affect uptake and retention.

We will generally do the observations without taking any detailed notes to avoid distraction. However, we will jot down important things that we can easily forgot during the course of observations even though we will keep this as minimal as possible. We will make detailed notes of the observations before the end of each field day while things are still fresh in the memory of the observer.
