# Supplementary material for: "I found that I was well and strong": Women’s motivations for remaining on ART under Option B+ in Malawi
Source: PLoS One. 2018 Jun 6;13(6):e0197854. doi: 10.1371/journal.pone.0197854 (PMC5991368; doi:10.1371/journal.pone.0197854)
Supplement: S3 Text — (DOCX) [file pone.0197854.s003.docx]

**“I found that I was well and strong”: Women’s motivations for remaining on ART under Option B+ in Malawi [PONE-D-17-27063] [EMID:379ee272e0420312]**

# **DATA COLLECTION TOOLS: PATIENT LEVEL BARRIERS TO RETENTION**

# **In depth Interviews Guide**

## **Socio-demographic data**

Collect the following socio-demographic details from all the participants at the beginning or at the end of the interviews.

- Age, marital status, number of children, ethnicity, highest education, area of residence (urban/rural), occupation, employment status, income and religion.

## **Context**

The following questions may help us to understand whether the woman is still in denial or has accepted her HIV status. This could be important in understanding LTF.

- When and where were you tested HIV positive?

***Probe****: who was with you?*

- How did you receive the news that you are HIV positive?

***Probe****: were you surprised? What were your worries?*

- How did the counsellor assist you on the day you tested HIV positive?

***Probe****: tell me what you like and what you did not like the most. What are the things they told you? Did you receive the ARVs the same day? Where did you get the ARVs?*

- How are you now? Any worries?
- How many people in your family know that you are HIV positive? What about friends?

***Probe****: what were their reaction? Do you regret informing them?*

- Why did/didn’t you disclose your HIV status to your family members/friends

***Probe****: if not yet ask when and how she is planning to tell them*

- How many children do you have? Do you use any family planning method?
- Did you plan for this pregnancy/child? Who wanted to have it most?

***Probe****: you, your husband, or both?*

## **Patient related barriers**

- You have been coming to the hospital to get medication (ARVs); do you know why these medicines are important to you?

***Probe****: where did you get the information?*

- What do you think about the medicines and its use?

***Probe****: do you think they help you or your baby? How?*

- When did you start the medication? Do you have any problem with the medicines?

***Probe****: do you experience any side effects? Have you ever stopped/skipped some days because of any of these problems?*

- I understood that you once stopped taking ARVs/coming to the facility to get more medicines. Do you mind sharing what happened? **(Ask only women who once defaulted)**

***Probe****: Ask for a story from the woman, reasons for defaulting, about her health when she stopped taking ARVs, and what made her to return to the clinic*

- What are the challenges you face to come to the clinic.

***Probe****: do you have any confidentiality concerns? Any issues at home? What are some of the issues that may make her consider stopping ART?*

- What motivates you to continue coming to the clinic

***Probe****: reasons for adhering to care*

## **Family barriers affecting the patient**

- Does your husband know about your HIV status?

***Probe****: If yes, how did he know? Why did you tell him? How did he react when you tell him about your status? Do you freely go to ART clinic or there are problems - explain*

- What kind of support do you get from your husband relating to ART?

***Probe****: is it enough? What else would you want him to do?*

- Are there times you have failed to go to the clinic because of issues relating to your husband? Explain
- Is there any other person who knows about your HIV status?

***Probe****: ask her to list them all and their relationship. Ask why she told each one of them.*

- What kind of support do you get from extended members of your family or friends?

***Probe****: is it enough? What else would you want them to be doing?*

- Have you ever failed to go to the clinic because of issues relating to your family members or friends?

## **Community barriers affecting the patient**

- Do you get any support from community members in general or from community-based organisations?

***Probe****: list the organisations and the kind of support they provide.*

- What are some of the issues in your community that motivates/demotivates you in regards to ART / PMTCT?

***Probe****: gossip, stigma, etc.*

- How is the environment in this community; is it conducive enough for you to go to the ART clinic freely? What challenges are there?
- What kind of support would you want to have from the community in general?

## **Health facility barriers affecting patients**

- How frequently do you go to the hospital?

***Probe****: Are you okay with that arrangement? What are the problems?*

- To which clinic do you go?

***Probe****: why do you go to that clinic? Is it the nearby clinic, if not ask for an explanation*

- How do you go to the hospital?

***Probe****: Do you walk or use public transport? How much does it cost you for transport, food and any other thing?*

- How many hours does it take to go to the clinic and comeback?
- How do they assist you at the hospital?

***Probe****: to which places do you go? How do they assist you in those places? What things do you like or dislike?*

- Do you have any confidentiality concerns with the facility set up or with the health workers?
- Are you satisfied with the way the health workers assist/treat you? Cite examples

***Probe****: Where do you think the health workers need to improve?*

- Have you ever decided or thought about defaulting because of the way health workers treated in the previous visits? Explain
- What do you think should be done to make our clinic a better place for everyone going for ART?

## **General barriers affecting patients**

- Do you seek any other type of help apart from the ART?

***Probe****: If yes, briefly explain each type of care, reasons for uptake, and if it is taking concurrently with ART*

- Is there any conflict between your religion and going to the clinic?

***Probe****: what does your religion say about HIV and ART?*

- Would you briefly explain the internal household process you have to follow when you want to go to the clinic

## **Home visits by Counsellor**

- Is there any counsellor visiting you home?

***Probe****: male/female? How frequent? Where do you meet? Do people ask about him/her? What do you tell them? Are you comfortable with his/her visits? If the husband is not aware of her status, probe what she tells him about the visitor.*

- How did you meet the counsellor and how did your relationship start?

***Probe****: how did they recruit you?*

- What do you discuss?

***Probe****: do you know why he/she visits you? How are you benefiting from him/her visits?*

**Thank you for your participation ☺**

# **Focus Group Discussions Guide**

## **Socio-demographic data**

- Assign numbers to all the participants and advise them to mention their number each time they are contributing to the discussion
- Collect the following details from each woman. Use the numbers above instead of names: Age, marital status, number of children, ethnicity, highest education, area of residence (urban/rural), occupation, employment status, income and religion.

## **Topics for discussions**

- You have been coming to the hospital to get medication (ARVs); do you know why these medicines are important to you?

***Probe****: where did you get the information?*

- What do you think about the medicines and its use?

***Probe****: do you think they help you or your baby? How?*

- Do you have any problem with the medicines?

***Probe****: do you experience any side effects? Have you ever stopped/skipped some days because of any of these problems?*

- What are the challenges you face to come to the clinic.

***Probe****: do you have any confidentiality concerns? Any issues at home? What are some of the issues that may make her consider stopping ART?*

- What motivates you to continue coming to the clinic
- When you learned that you are HIV positive, how did you react?

***Probe****: what came first into your mind? What did you do when you went home?*

- What support did you receive from the counsellor?

***Probe****: were you satisfied? What do they need to improve?*

- What is the perception of people in our communities towards people living with HIV?

***Probe****: are you accepted? Are you able to tell people about your status?*

- What kind of support is there for people with HIV in your communities?

***Probe****: all organisations, what they do and if they are useful to them.*

- Is there any counsellor visiting you home?

***Probe****: male/female? How frequent do you meet and where? Do people ask about him/her? What do you tell them? Are you comfortable with his/her visits (explain)? If the husband is not aware of your HIV status, what do you tells him about the visitor.*

- How did you meet the counsellor and how did your relationship start?

***Probe****: how did they recruit you? Were you happy? What need to change?*

- What do you discuss?

***Probe****: do you know why he/she visits you? How are you benefiting from his/her visits?*

- I am sure there are times we fail to come to the clinic for our appointments. Can you share with us what are some of the key issues that make us to honour our appointments?

***Probe****: Ask for reasons and number of appointments missed in the last 6 months*

- What are the challenges or problems that hinder access to ART clinic
- Does your husband know about your HIV status?

***Probe****: If yes, how did he know? Why did you tell him? How did he react when you tell him about your status? Do you freely go to ART clinic or there are problems - explain*

- What kind of support do you get from your husband relating to ART?

***Probe****: is it enough? What else would you want him to do?*

- Are there times you have failed to go to the clinic because of issues relating to your husband? Explain
- Is there any other person who knows about your HIV status?

***Probe****: ask her to list them all and their relationship. Ask why she told each one of them.*

- What kind of support do you get from extended members of your family or friends?

***Probe****: is it enough? What else would you want them to be doing?*

- Have you ever failed to go to the clinic because of issues relating to your family members or friends?
- Do you seek any other type of help apart from the ART?

***Probe****: If yes, briefly explain each type of care, reasons for uptake, and if it is taking concurrently with ART*

- Is there any conflict between your religion and going to the clinic?

***Probe****: what does your religion say about HIV and ART?*

- Would you briefly explain the internal household process you have to follow when you want to go to the clinic
